# Effect of Isolation On Mental Health Profiles of Caregivers of Children with Medical Complexity: A Mixed Methods Embedded Case Study

**DOI:** 10.1155/ijpe/1003571

**Published:** 2026-03-10

**Authors:** Nora Fayed, Apsara Ali Nathwani, Rachel Martens, Eyal Cohen

**Affiliations:** ^1^ School of Rehabilitation Therapy, Queen’s University, Kingston, Ontario, Canada, queensu.ca; ^2^ Child Health Evaluative Sciences, The Hospital for Sick Children, Toronto, Ontario, Canada, sickkids.ca; ^3^ Azrieli Accelerator, University of Calgary, Calgary, Canada, ucalgary.ca; ^4^ Institute of Health Policy, Management and Evaluation, University of Toronto, Toronto, Ontario, Canada, utoronto.ca; ^5^ Department of Paediatrics, University of Toronto, Toronto, Ontario, Canada, utoronto.ca; ^6^ Edwin S.H. Leong Centre for Healthy Children, University of Toronto, Toronto, Ontario, Canada, utoronto.ca

**Keywords:** caregiver, case-study, children with medical complexity, mental health, mixed-method

## Abstract

**Background:**

The aim was to obtain mental health profiles of caregivers of children with medical complexity (Co‐CMC) during periods of adjustment and adaptation through embedded case study during shared isolation in 2020.

**Methods:**

A sequential mixed‐methods embedded case study of mental health adjustment from an online community‐dwelling sample of Co‐CMC, Canada. The *quantitative* ‘unit of analysis’ was collected prospectively to classify study participants as having ‘well’ or ‘below‐average’ mental health, based on norm‐referenced self‐reported mental health trajectories using the PROMIS General Mental Health Scale. The *qualitative* ‘unit of analysis’ was conducted with a purposively selected subgroup of Co‐CMC caregivers, from each trajectory group, to provide an inductive framework of mental health adjustment. Distinguishing features between caregivers who demonstrated well, from below‐average mental health, were obtained through triangulating both units of analysis into ‘profiles’.

**Results:**

Over 70% of Co‐CMC (n = 35) reported below‐average (i.e., unwell) mental health, while caregivers with average or above average mental health (i.e., well) (n = 12) showed group declines after 6 months. Home care choices, positive orientations, familial support‐adaptations, and adapted employment qualitatively distinguished members of the ‘well’ group from the ‘unwell’, while financial concerns were unique descriptions to Co‐CMC in the ‘unwell’ group.

**Conclusion:**

This study was the first in‐depth profile of mental health of Co‐CMC, based on prospective longitudinal data and qualitative descriptions to enhance understanding of the trajectories. The distinguishing factors can be used to screen and potentially prevent caregiver mental health problems, as well as identify interventions to promote thriving families.

**Conclusions**

• In‐depth prospective mixed method mental‐health profiles of Co‐CMC in the community showed ‘well’ versus ‘below‐average’ profiles were consistent with clinical samples from extant research
• Qualitative reports that distinguished the ‘well’ group from others were: positive caregiver outlook, family‐centred home care, familial support, accommodated employment, and sufficient finances.
• Although the ‘well’ group was able to thrive in the first 7 months of isolation, both Co‐CMC groups collapsed into the ‘below‐average’ range showing the time‐limited nature of positive caregiving in the context of isolation

## 1. Introduction

Children with medical complexity (CMC) are a small group [1], <1% of all children [2], often defined by their need for extensive healthcare service and caregiving [3]. Primary caregivers of children with medical complexity (Co‐CMC) manage their children’s health and development with life‐sustaining medical technologies [4] and educational supports [5]. Caregivers attempt to meet their own needs for respite with homecare and night nursing services [6, 7]. Studies have consistently reported higher proportions of mental health sequelae [7] and lower mental wellbeing among Co‐CMC compared to other parents their age [8–10] due to high caregiving demands [3], adaptation [4], financial hardship due to high medical costs [4, 7], strained employment [4], and inadequate homecare [7, 11]. Child‐specific characteristics empirically associated with caregiver mental health variations have been: the quantity of the child’s medical technology, level of physical impairment, and the child’s own emotional difficulties [10]. The presence and timing of these stressors have been unique to each family and their context, making it difficult to study the effects on associated factors, on caregiver mental health.

In 2020, isolation to members of one’s household was recommended by public health agencies [12] in most jurisdictions in Canada, while access to in‐person health‐care and education, was limited. The extant literature could not provide clarity as to which caregivers would thrive and which were at risk of mental health decline because in‐depth qualitative experiences had not been combined with prospective quantitative profiles of mental health, as is shown in mixed method case‐study research. Thus, stressors were similar for all caregivers (household isolation), but the way in which they thrived or succumbed to mental health difficulty in response, provided an opportunity to observe the differential effects of strain to an already vulnerable group.

Psychological profiles are clinically useful for describing mental health groups and subgroups. An inductive and descriptive approach to generating profiles can be useful for identifying vulnerable or resilient Co‐CMC during disruptive periods, because they can incorporate more broadly defined considerations than those that have been previously described. In tandem, standardized classification of mental health status is important to comparing and contrasting ‘well’ or unwell (below‐average) groups in an objective way. The purpose of this study was to obtain profiles of Co‐CMC mental health in response to life disruptions, through mixed‐method case‐study of the first calendar year of the pandemic (April‐December 2020), when the antecedent stressors of isolation, schooling, and homecare disruptions, were at their societal peak [13, 14].

### 1.1. Objective

Obtain Co‐CMC ‘mental health profiles’ of wellness or vulnerability through descriptive case‐study of an on‐line community dwelling sample during 9 months of shared isolation.

### 1.2. Methods

**Figure 1:**
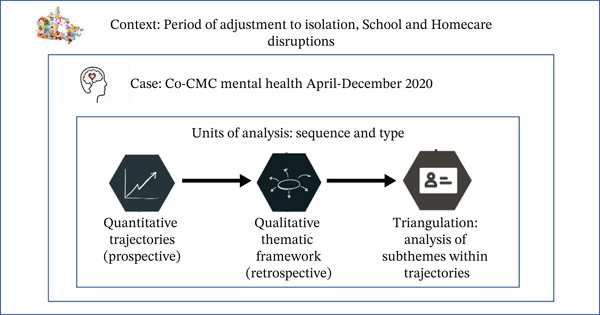
Overview of sequential mixed‐method embedded case study to obtain Co‐CMC mental health adjustment profiles.

Approval was obtained through The Health Science Research Ethics Board (#REH‐805‐21) from Queen’s University, Canada. The study design was a mixed‐methods sequential, (quantitative to qualitative) [15], embedded case study [16] of the mental health of Co‐CMC, from on‐line community‐dwelling convenience sample (Figure 1). The ‘quantitative unit of analysis’ was collected prospectively using monthly Co‐CMC self‐reported mental health, to descriptively classify participants as having ‘well’ (above‐average or average) vs ‘unwell’ (below average), trajectories. Trajectory classification was based on norm‐referenced self‐reported mental health over 9‐months, using larger tertiary‐care samples of Co‐CMC with identical eligibility criteria, using previously validated mental health trajectories [10],. The ‘qualitative unit of analysis’ was obtained through follow‐up 1:1 interviews, at the end of the ‘quantitative unit of analysis data‐collection period’, of a purposively sampled subgroup of Co‐CMC from the classified ‘well’ versus ‘unwell’ trajectory groups. Triangulation of both ‘units of analysis’ into ‘profiles’ was planned through between‐trajectory sub‐theme analysis, that distinguished ‘well’ from ‘unwell’ caregivers. A preprint was previously been published [17].

#### 1.2.1. Eligibility and Recruitment

On‐line screened caregivers of children, who satisfied the definition for CMC were invited to participate, if: (i) they were a caregiver for a medically complex child who met an operational definition of eligibility for structured complex care programs (technology‐dependent, high health use, chronic >6 months) [1, 4], ii) lived in Canada, and able to iii) read and speak English or French.

#### 1.2.2. Setting

Community dwelling convenience samples of Co‐CMC on social media (e.g., Facebook and ‘X’) were the study target, which were further disseminated by the patient‐engagement expert from the study team online.

#### 1.2.3. Outcome Measure

Mental health was assessed using the 5‐item subscale of the PROMIS‐Global 10 v1.2, General Mental Health Scale [18]. The measure generated a T‐score of each caregiver’s mental health where a mean of T = 50 represented equivalence with other adults of the same age and sex from the general population. Each 10 points above or below 50, represented one standard deviation above or below the population average. For example, a T‐score of 35 indicated, that an individual was 1.5 standard deviations below the population mean, equivalent to a self‐reported mental health that is lower than 93% of adults of the same age and sex [18]. This provided a substantively relevant estimate of a caregiver’s mental health each month.

Measures were administered monthly, for 9 months. Lack of completion of the survey within 30 days of the last entry was considered missing data. Only participants with at least 3 months of mental health data were included in the trajectory analysis.

##### 1.2.3.1. Quantitative Unit of Analysis: Mental Health Trajectories.

Descriptive statistics of the sample included child, caregiver and household characteristics at baseline. Individual mental health scores were plotted serially and classified using Kazdin’s visual analysis of data for case studies [19], against previously validated Co‐CMC mental health trajectories in a pre‐Covid sample using the same measure [10]. The classification using visual analysis and scores was completed by 2 researchers, with expertise in Co‐CMC mental health (AAN and NF), first independently, then collaboratively, over three iterations, using the criteria of mental health trajectories from a larger, pre‐Covid Co‐CMC sample [10] of: i) magnitude of mental health T‐score serial plots (relative to population norms) and ii) shape (e.g., increasing, decreasing, 0‐slope, linear/curvilinear). This classification with visual analysis approach has been validated as concordant to statistical tests on larger data sets for medium and large effect sizes [20].

##### 1.2.3.2. Qualitative Unit of Analysis ‐ Co‐CMC Mental Health Adjustment and Adaptation.

An interpretive descriptive approach was used to construct a framework of the caregivers’ mental health [21]. Participants were purposively sampled from the ‘well’ and ‘unwell’ quantitative trajectory groups, respectively, and interviewed until data saturation was obtained. A semi‐structured interview guide was employed to elicit mental health narratives, their own mental health perspectives, and any events or attributions they reported to have affected them.

Two analysts (NF and AAN) iteratively and inductively coded the data into (sub‐themes) and components (themes) using thematic analysis and analytic structure of Braun and Clarke’s (2006) phases of analysis [22], constant comparison, and an interpretive lens, as researchers of caregiver mental health for CMC.

##### 1.2.3.3. Triangulation ‐ Mental Health Profiles.

The final aim, to obtain Co‐CMC mental health profiles, was accomplished through a more granular analysis of qualitative subthemes between each trajectory group. For example, if a theme of ‘caregiving‐support’ emerged from the data, with a subtheme about ‘home‐care’, an analysis of all the codes and quotes that formed ‘home‐care’, would be conducted separately for ‘well’ in contrast to ‘unwell’. Sub‐theme findings that were unique to a group, were noted as ‘distinguishing’ or ‘profile characteristics’.

## 2. Results

### 2.1. Quantitative Unit‐Mental Health Trajectories

**Figure 2:**
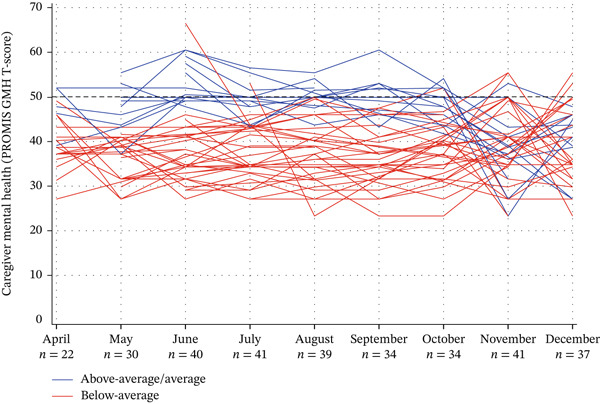
Individual Co‐CMC trajectories (N = 46) classified as average/above‐average (well) mental health and below average (BA) mental health based on Nathwani et al. (2024) mental health classifications and Kazdin’s visual analysis method, using PROMIS General Mental Health T‐Scores.

Table 1 presents the baseline characteristics of Co‐CMC, categorized by two mental health groups identified from 46 Co‐CMC who completed >3/9 months of data collection. The individual trajectories of both groups i.e., those with average/above‐average mental health (well) and those with below‐average mental health are shown in Figure 2. In the first 6‐7 months of isolation, distinct trajectories were observed for ‘well’ and ‘well’ groups, while months 8 and 9, marked a change, in that individuals from both groups, converged towards a below‐average mental health status.

**Table 1:** Participant characteristics. All values expressed as n (%) unless otherwise stated.

	Caregiver Mental Health N = 46	
	Average/Above‐Average (well) Mental Health Groupn = 12 (26%)	Below Average (unwell) Mental Health Groupn = 34 (74%)
*Caregiver Characteristics*		
Age, y		
*Mean (SD)*	40 (5.5)	41 (6.5)
Female	12 (100)	34 (100)
Caregiver type		
*Mother*	11 (91.7)	33 (97.1)
*Other*	1 (8.3)	1 (2.9)
Education		
*Secondary/high school*	0	4 (11.8)
*Some post-secondary (University/college)*	1 (8.3)	6 (17.6)
*University/college*	11 (91.7)	24 (70.6)
Employment		
*Employed (full or part-time)*	7 (58.3)	11 (32.4)
*Full-time caregiver (stay at home parent)*	4 (33.3)	23 (67.7)
*Student*	1 (8.3)	0
*Child Characteristics*		
Age, y		
*mean (SD)*	8 (3.0)	7 (5.5)
Sex		
*Female*	5 (41.7)	14 (41.2)
*Male*	7 (58.3)	20 (58.8)
Pre‐COVID‐19, Child Was Attending In‐Person School or Daycare	10 (91)	20 (57)
*Household Characteristics*		
Family Structure		
*Married or living common-law*	11 (91.7.9)	29 (85.3)
*Single, separated, widowed or divorced*	1 (8.3)	5 (14.7)
Number of children in the home *mean (SD)*	2 (1.6)	2 (1.0)
# of family‐caregivers providing regular care		
*1*	0	8 (24)
*2*	12 (100)	25 (76)
Annual Household Income, dollars) ^∗^		
*<20* k*-40* k *(poverty-level income)*	2 (16.7)	7 (20.6)
*Between 40-60* k *(below average)*	0 (0)	2 (5.9)
*Between 60-80* k *(below average)*	0 (0)	3 (8.8)
*Between 80-100* k *(mean income range)*	3 (25)	6 (17.6)
*>100 k*	5 (41.7)	,11 (32.4)
*Missing*	1 (8.3)	5 (14.7)
*Distance from Facility*		
Distance (in km) to the nearest medical facility specialized for child’s medical technology needs		
*median (IQR)*	23 (10‐81)	31 (20‐150)
*Healthcare Expenses*		
Out‐of‐pocket spending on medical technology, in dollars ^∗∗^		
*Median (IQR)*	100 (100‐2000)	800 (100‐5000)
*Missing*	1 (8.3)	15 (44.1)
Out‐of‐pocket spending on medicines, in dollars ^∗∗^		
*median (IQR)*	250 (0‐12000)	300 (0‐1500)
*Missing*	1 (8.3)	13 (38.2)
Private Health Insurance ^∗∗^		
*Yes*	11 (91.7)	29 (85.3)

^∗^Based on average low‐income cut‐off values from Statistics Canada, https://www150.statcan.gc.ca/income_2020_21. Expressed in Canadian dollars.

^∗∗^Expressed in Canadian dollars.

^∗∗∗^Canadian private insurance covers ‘non‐essential’ health services according to the Canada Health Act, in some territories, this includes child’s medication and equipment.

### 2.2. Qualitative Unit ‐ Co‐CMC Mental Health Framework

Seven core themes of mental health are outlined in Table 2, as well as any sub‐themes that ‘distinguished’ between the ‘well; and ‘unwell’ groups.

**Table 2:** Co‐CMC mental health adjustment and adaptation framework themes and subthemes divided into distinguishing versus non‐distinguishing subthemes.

Theme	Subtheme Found to be Distinguishing Between Well and Unwell Groups (n = 12)	Subtheme with Common/Indistinguishable Findings Between Well and Unwell Groups (n = 13)
I ‐ Child Wellbeing	Concerns about current development, mental, social and physical health	Concerns about future development, mental, social, and physical health
II ‐ Caregiver (Co‐CMC) Mental Health	General mental health	Specific emotions (e.g., anger, exhaustion)
	Health hygiene habits	Covid‐19‐specific emotions (e.g., fear of the virus)
	Socializing and relationships	Mental health history and symptoms of mental disorder
		Lifestyles
		Major life events (e.g., death in the family)
		Worries about the future
III – The Caregiver’s, Decisions & Demands	Choices or lack of choices about homecare	
	Caregiver roles and tensions	
	Employment	
IV ‐ Supports & Barriers	Homecare and respite	Ambulatory care access
	Child school	Allied health support
		Access to health care
		Support from family and community
V ‐ Material Needs	Finances	Supplies (e.g., medical technology, PPE, drugs, and formula)
		Housing
VI ‐ Societal Context	Covid‐19 information	
	Reactions to community and government pandemic responses	

Theme, I, ‘Child Wellbeing’ = Co‐CMC concerns and observations about their medically complex child’s physical and socio‐emotional health, development and wellbeing during the pandemic and for the future. Theme II, ‘Co‐CMC Mental Health’ = the mental health history emotions, emotional states, and habits and events that shaped their mental wellbeing or lack thereof. Theme III, ‘Scope of Caregiving Decisions and Demands’=the caregiver’s strategies, roles and responsibilities, in which multiple domains of life were described, ranging from those within the direct of control of the caregiver, to external pressures or demands placed upon them. Theme IV, ‘Supports and Barriers’= formal and informal support from family, community, health care and the child’s education providers were described by caregivers as enabling or generating barriers to their mental health. Theme V, ‘Material Needs’ = stress or ease of access to medical supplies, financial issues, and housing. Finally Theme VI = ‘Covid‐19 Societal Context’ referred to the effects of Covid‐specific government responses, public‐health policies, and media on the mental health and wellbeing of the caregiver.

### 2.3. Triangulation/Profiles of ‘Well’ (Above‐Average and Average) vs. ‘Unwell’ (Below‐Average’) Mental Health

**Figure 3:**
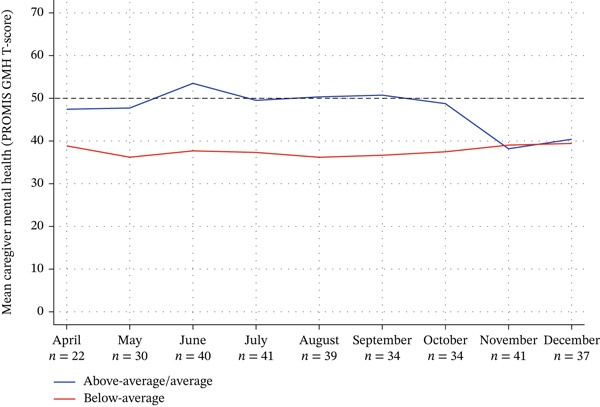
Mean Co‐CMC mental health trajectories of cases from average/above‐average (well) mental health and below average (unwell) mental health groups using PROMIS General Mental Health T‐Scores.

The quantitative strand of data showed that only in the first 7 months of the pandemic, mental health scores between cases from well and unwell groups demonstrated distinctions, the mean trajectories of which were shown in Figure 3.

Although both well and unwell cases demonstrated variable mental health scores, with trajectories that were neither consistently increasing or decreasing, ‘well’ cases rarely fell below 1SD below the mean (i.e., T = 40), while unwell cases were almost always less than the mean of T = 50. Participants provided their interpretation of these trajectories:

*“My mental health is really a reflection of how things are going with my kids…And, actually, it was very stable. This is what it shows. It was almost a perfect straight line, I’d say.” -ID 23.*

In the months of November and December, a convergence of mental health scores occurred into the below average range (<T = 40), were all caregivers from both groups showed challenges (Figure 1). Reports of this phenomenon was explained:

*“And I think now is when the mental health issues are starting to like emotionally flood on to you that you try to process stuff that you haven’t processed, right?” -ID 28*

**Theme.**

**I**, ‘Child Wellbeing’, caregivers from the unwell group, highlighted worries about the effect of the pandemic on their children:

*“it’s hard for him because everybody’s next level gowns and masks and goggles. If he could just be treated like other kids … that would probably improve how I feel about things”. -ID 100.*

In **theme II,** *all* Co‐CMC reported that they experienced negative emotional experiences, but members of the well group also mentioned positive or ‘silver‐linings’ perspectives about their:

i. overall mental health:

*“I actually think I’m a better parent than before COVID. I started out feeling all crushed and panicky and stuff, but then as we adjusted to our new normal, I feel like I’m better off than I was before.” -ID 23*

ii. … or their mental health hygiene and habits:

*“I developed some skills and took part in some programs and started medication and got more active again. I have had periods in my life earlier than that where I did try to have a relationship with a higher power. This one was better. This time it worked.” -ID 106*

or, iii) the ability or good fortune too maintain healthy socializing:

*“Luckily my parents live within 45 minutes, so they bubbled with me, so I spent a lot of time with them this year, which has also been great. So yeah, there’s – that’s the other benefit.” -ID 95.*

Thus, positive perspectives and experiences of overall health, habits, and social relationships were found to be distinguishing characteristics, of a positive mental health trajectory.

The theme with the most granular details, ‘Caregiver Decisions and Demands’, **theme III,** was composed of three sub‐themes that were experienced differently by members of the well group in contrast to unwell. Well members reported i) having *choices* about homecare that were tailored to their needs, ii) *adapting* to performing new or strained roles, and iii) *receiving accommodations* from their employers.

Having homecare choices was exemplified by a ‘well’ member here:

*“We just gave all of our nurses a choice, like just choose wherever you want to choose but we can’t have people working in multiple locations because we didn’t feel safe with that… and then three of them decided to just work for us which worked well”. -ID 17.*

In contrast, members of the ‘unwell’ group highlighted feelings of helplessness about their homecare:

*“Having funding through health [publicly], decreased our control because we were depending on systems that have historically failed to provide us with homecare. But also, we’re in a system-wide crisis around human-resource shortages. The pandemic restrictions also decreased our control over access. We had no control over any of that.” -ID 144.*

Co‐CMC reactions to the multiple roles and the tensions placed on them also differed between trajectory groups, such that the well group coupled their guilt with self‐forgiveness about work‐caregiving tensions, while the unwell group described dominant feelings of guilt, and unmanageable and or grueling caregiving.

*“The major guilt is, should I even be working with a child at home? You know, am I doing a disservice to my employer by accepting their pay for half the work? You know what I mean? Like there’s all these types of guilt that aren’t necessarily in your control, but your mind plays games on you, that, you know, you’re a horrible mother because you’re not spending enough time with your kid, or you’re a horrible employee because you’re not dedicating enough time for your work.” -ID 28.*

Overall, in theme III, having agency over caregiving and caregiving supports distinguished between Co‐CMC from unwell and ‘well’ groups.

In **theme IV**, “Supports and Barriers’ the experiences of ambulatory care, specialized allied health, and even family support were important to both groups. Individuals from ‘well’ and ‘unwell’ both described mixed (positive and negative) experiences of these supports. However, experiences of homecare, respite and the child’s schooling to Co‐CMC mental health in the early part of the pandemic were shown to distinguish cases from ‘well’ relative to unwell. For example, this person from ‘well’ reported feeling family‐centeredness, adequate levels of support, and ability to fund their own supports.

*“The other things that were helpful was having like really positive relationships with each member of our nursing staff and being able to have like open and transparent conversations with them … we can only offer like two staff members fulltime hours… [Our] great home care support made not having the same social supports a little bit easier to manage.” -ID 17.*

Unwell families in particular felt unsupported in their decision‐making:

*“Shared decision making is support. And leaving me on my own to make my own decisions when I feel I don’t have enough information to make good decisions, is a form of abandonment.” -ID 106.*

The caregiver’s experience of the child attending school, not attending, or engaging in some form of homeschool, also appeared to distinguish cases between trajectory groups. Contrast for example this caregiver’s experience from the well group:

*“[my son] not being in school has had so many positives that we may not go back to having him go in person to school.” -ID 23.*

… to this mother from the ‘unwell’ group’s concerns about her child:

*“She is not in school because there’s a nursing shortage. So, I’m trying to find regular preschools for her and they’re like, [We don’t know what to do if something happens to that G-Tube].” -ID 130.*

Overall, caregivers who reported wanting or needing homecare and schooling that they did not receive, were represented in the unwell group.

In **theme V** ‘Material Needs’, the procurement of necessary ‘supplies’ (including medical technology replacement parts, personal protective equipment, medication, and special food or formula), was sometimes met and other times challenging for cases *in both* mental health groups. Types of ‘housing’, (e.g., apartment, free‐standing home, etc) did not distinguish the mental health of either group, noting however, no participants reported unstable housing. Co‐CMC from ‘well’ group reported they adapted to, or managed with, the finances they had, while those from the ‘unwell’ group reported they were often at their financial limits.

In the **theme VI** of ‘Societal Context’, cases from the ‘unwell’ mental health group reported feeling negatively affected by ‘Covid‐19 information’ that frightened them, or did not meet their needs for individualized information about *their* child. In contrast, members of the ‘well’ group reported they felt they could trust information from, their complex care providers. In the subtheme of ‘reactions to community and government pandemic responses’, cases from the unwell group reported feeling forgotten, marginalized, or left behind by their communities or society, while people from the well group reported positive societal reactions, in addition to the negative ones:

*“The goodwill of individual people [was what helped], as opposed to good planning or execution by the system.” -ID 144.*

## 3. Discussion

This study is the first of which we are aware to provide profiles that *distinguish* which caregivers’ traits and circumstances allow them to thrive or not, using prospective mental health trajectories *and* qualitative findings. Consistent with earlier research of Co‐CMC using the same measure, two‐thirds of our sample showed below‐average mental health [9, 10], conceptualized as self‐reported emotional and cognitive functioning, relative to population norms of adults of the same gender and age, from the general population [18, 19]. Yet our findings emphasized the importance of caregivers’ *choices* about formal supports such as homecare and schooling, to mental health, not the actual *amounts* of homecare, or in‐person schooling. Wishes for more or less homecare or schooling, were driven by combinations of factors unique to each caregiver, such as secondary caregivers in the home, additional caregiving roles (e. g., to other ill family or work), and finances [23, 24]. The emphasis on family‐centered and shared decision‐making about homecare for Co‐CMC and their children, implied that empowered experiences of homecare would be a better predictor of good outcome for future research, and should be included with other medical home standard [25] quality indicators.

A positive outlook, or caregiver reports of ‘silver‐linings’, seemed to distinguish between the Co‐CMC who showed ‘well’ mental health in the first 6 months of pandemic stress, from those who did not. Co‐CMC who talked about familial cooperation and employment accommodations, had adapted their routines, formed stronger intra‐familial relationships, and healthy habits, appeared to have mitigated the challenges of isolation or disrupted formal supports. Such results were in alignment with extant literature on caregiver resilience and coping [26, 27] and lend further support to strength‐based interventions^28-30.^ Caution should be exerted recommending ‘positive orientations’ as an intervention goal in and of itself, especially in the absence of family‐centred supports, because it remains unclear whether such orientations are a consequence of good supports, or vice versa.

Positive adaptations of ‘well’ caregivers, may have ultimately been insufficient to counter the consequences of lengthy adversity as shown by the convergence of poor scores toward the concluding months of the data collection period at the end of 2020, and the qualitative unit of analysis supports this interpretation. The causality for the convergence between groups in the below range cannot be ascertained, only described. The time‐limit associated with positive caregiving, appears to be a non‐Covid‐specific finding, because in wider literature, the mental health of above‐average individuals has been shown to be more likely to return to baseline following life‐disruptions [28–30]. The implication from our findings is, that positive caregiver orientation is a precious resource, with time and circumstantial limits.

The caregivers outlined that ambulatory care, rehabilitation, and dietetic, social work, medical technology supplies, and housing [4, 31–33], were important, but they did not appear to distinguish good or bad outcomes during this period. In general, our trajectory groups were limited by a small sample of respondents, a challenge to obtain, for this already small and burdened population. The representativeness of the sample is inconclusive, for example, it appeared that there were a higher proportion of individuals living further away from their main providers (i.e., rural) and with lower household incomes than previous clinical samples [10]. However, this community sample appeared to have similar mental health distributions to clinical ones [10]. More comprehensive study of Co‐CMC should include larger cohorts for longer periods to determine the sequence and timing of factors that will positively impact long‐term health for them and their children.

## Funding

Social Sciences and Humanities Research Council of Canada, 10.13039/501100000155, 430‐2020‐00613; SickKids Foundation.

## Ethics Statement

Approval was obtained through The Health Science Research Ethics (#REH‐805‐21) from Queen’s University, Canada.

## Conflicts of Interest

No, there is no conflict of interest. The funders of public research declared were not involved in the conduct, interpretation or dissemination of the research.

## Data Availability

The data that support the findings of this study are available on request from the corresponding author. The data are not publicly available due to privacy or ethical restrictions.
